# Differential Bacterial Colonization and Biofilm Formation on Punctal Occluders

**DOI:** 10.3390/ma12020274

**Published:** 2019-01-16

**Authors:** Michael Hadjiargyrou, Eric D. Donnenfeld, Lola M. Grillo, Henry D. Perry

**Affiliations:** 1 Department of Life Sciences, New York Institute of Technology, Old Westbury, NY 11568, USA; 2 Ophthalmic Consultants of Long Island, 711 Stewart Avenue, Suite 160, Garden City, NY 11530, USA; ericdonnenfeld@gmail.com (E.D.D.); hankcornea@gmail.com (H.D.P.); 3 Nassau University Medical Center, East Meadow, NY 11554, USA; lola.grillo@gmail.com

**Keywords:** punctal, occluders, plugs, biofilm, *S. aureus*, bacteria, dry eye disease

## Abstract

Dry eye is a common condition that is treated primarily by topical lubricants, immunomodulation, and a variety of punctal and canalicular plugs (occluders). Biofilm formation has been reported as an ongoing problem with the clinical use of occluders. In order to explore the role of biofilm formation on occluders, we tested the bacteria strain, *Staphylococcus aureus*, with three different types of occluders, Delta^R^, Odyssey^R^, and Alphamed^R^. Scanning electron microscopy (SEM) of these occluders revealed a variation in surface appearance, with Odyssey^R^ being the smoothest (but with grooves), followed by Delta^R^, and Alphamed^R^. Exposing each type of occluder to dynamically grown bacterial cultures of *S. aureus*, a ~3 fold statistically significant difference in bacteria colonization between the Odyssey^R^ and Alphamed^R^ occluder and a ~2 fold higher trend between Odyssey^R^ and Delta^R^ were detected. These quantitative results were also verified with SEM, showing extensive *S. aureus* colonization and biofilm formation on the surface of the Odyssey^R^ occluder. The results also indicate that bacterial colonization readily occurs on all three types of occluders. The occluder with the smoothest but grooved surface (Odyssey^R^), displayed increased biofilm formation when compared to those with rougher surfaces.

## 1. Introduction

Dry eye disease (DED) is arguably the most common ocular disease presenting to the eye care professional, affecting approximately one of every four patients in an ophthalmologist’s office. As a progressive condition, and if left without treatment, it can lead to vision loss, ocular surface damage, discomfort, and overall reduction in quality of life [1,2,3]. DED is treated primarily by topical lubricants, immunomodulation with cyclosporine, lifitegrast and corticosteroids, oral nutrition with omega-3s, antibiotics, lid hygiene, and a variety of punctal and canalicular plugs (occluders) [1,2,3]. Punctal occluders are particularly effective in patients with aqueous insufficiency and DED, because they prevent outflow of tears through the punctum and preserve the natural tear lake [4]. Punctal occluders can be permanent, with an exposed flange that rests on the surface of the punctum, or temporary and dissolvable when placed within the punctum [5].

Biofilm formation has been reported in the literature as an ongoing problem with the clinical use of occluders, leading to infection, inflammation, and intolerance [6]. First coined by William J. Costerson in 1978, the term “biofilm” describes “surface-attached microbial agglomerations”, or a capsular polysaccharide enabling bacteria to attach to a device [7] Within the biofilm, bacteria produce extracellular polymeric substances (EPS) that include extracellular polysaccharides, proteins, lipids, and DNA. One of these proteins is adhesin, which facilitates the adhesion of the biofilm to a surface, which, in turn, prevents the colony from being dislodged into the environment [8]. The scaffold of the biofilm not only insulates bacteria from anti-infectives, disinfectant systems, and even our own white cells, but also facilitates further bacterial adhesion and colonization [7,8,9,10]. *S. aureus* is an excellent example that demonstrates this [11]. Our study examined the formation of biofilms in vitro on three popular silicone-based punctal occluders using *S. aureus*. This strain was chosen because *S. aureus* is the most common bacteria found on punctal occluders [12], and also because it is known for biofilm formation on ophthalmic devices [13]. In addition, *S. aureus* can also cause corneal ulceration [14]. Dynamic microbial cultures, histological staining, and SEM were all used to investigate the bacterial colonization and biofilm formation on the three types of occluders.

## 2. Materials and Methods

### 2.1. Bacteria Strains

The bacterial strain, *Staphylococcus aureus* (#10390), was purchased from American Tissue Culture Collection (ATCC, Manassas, VA, USA). Bacteria were streaked on nutrient broth agar plates and incubated overnight at 37 °C. A single colony was selected and grown in 3 mL of nutrient broth overnight in a shaking 37 °C incubator to establish the liquid cultures.

### 2.2. Punctal Occluders

**Figure 1:**
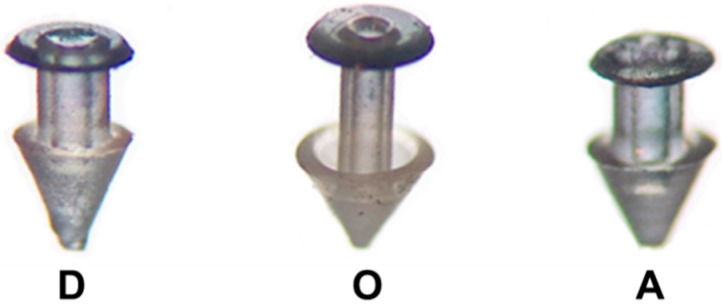
Morphology of occluders. The three types of occluders used in the study were: Delta^R^ (D), Odyssey^R^ (O) and Alphamed^R^ (A). The top diameter of the three occluders was 0.9 mm and their length ~2 mm.

Three different types of punctal occluders were used in this study from the following manufacturers: Delta Life Sciences^R^ (Comfortear^R^), Odyssey Medical^R^ (Parasol^R^), and Alphamed^R^ (Quintess^R^). All were hydrophobic silicone-based and measuring 0.9 mm in greatest diameter (Figure 1) [5]. The length of each occluder was approximately 2 mm. Light microscopy was performed on all three punctal occluders and all three have a flat head, a thin neck, a thick cone-shaped base, and a central lumen (Figure 1).

### 2.3. Occluder Exposure to Bacteria

Each occluder type (n = 4 for each type) was placed individually in 5 mL of nutrient broth containing growing *S. aureus* cells. The culture were placed in a shaking 37 °C incubator. Every morning, for the next seven days, 5 mL of the growing bacteria were removed and replaced with an equal amount of fresh nutrient broth so that the existing bacteria would continue to grow. The total time that all occluders were exposed to the bacteria was seven days.

### 2.4. Bacteria Biofilm Quantitation

Following seven days of continued exposure to the dynamically growing bacterial cells, the occluders (n = 3 for each type; another one was used for SEM, see below) were removed from the cultures and placed in a microcentrifuge tube. Following three washes with distilled water (300 μL each time), 50 μL of 0.1% crystal violet was added, and then incubated overnight at Room Temperature (RT). The same procedure was done with unused (n = 3 for each type). Following staining, the crystal violet was eluted by rinsing the stained occluders three times with distilled water (300 μL each time), followed by the addition of 95% ethanol (20 μL) and incubation for 15 min at RT. Finally, the eluted crystal violet from each occluder (both experimental and unused control) was measured in a spectrophotometer at an OD (Optical Density) of 600 nm. The OD from the control samples was subtracted from the experimental values before the data was plotted.

### 2.5. Scanning Electron Microscopy (SEM)

The presence of bacteria and overall biofilm formation on each of the three different types of occluders was evaluated by SEM. Prior to SEM, each occluder was washed three times (5 min each time) in 1× Phosphate Buffer Saline (PBS). The occluders were then dehydrated serially in alcohol washes, as follows: 70%, 80%, 90% (15 min at RT) and 100% (three times for 15 min at RT). Images were obtained on a LEO 1550 Field Emission Scanning Electron Microscope (Carl Zeiss AG, Oberkochen, Germany), with a Schottky Field Emission Gun, at an acceleration voltage of 20 kV and a Robinson Back Scatter Detector. Occluders were gold coated to prevent charging effects.

### 2.6. Statistical Analysis

For the spectrophotometric data of bacterial colonization/quantification, each type of occluder was tested in triplicate and the results are presented as group mean +/− standard deviation (SD). The significance of the results was determined using the Kruskal–Wallis nonparametric ANOVA for multiple comparisons. The significance for all tests was p < 0.05. The statistical software package SPSS 22 for Windows (version 22, SPSS, Chicago, IL, USA) was used for data analysis.

## 3. Results

### 3.1. Variation of Bacterial Growth on Different Occluders

**Figure 2:**
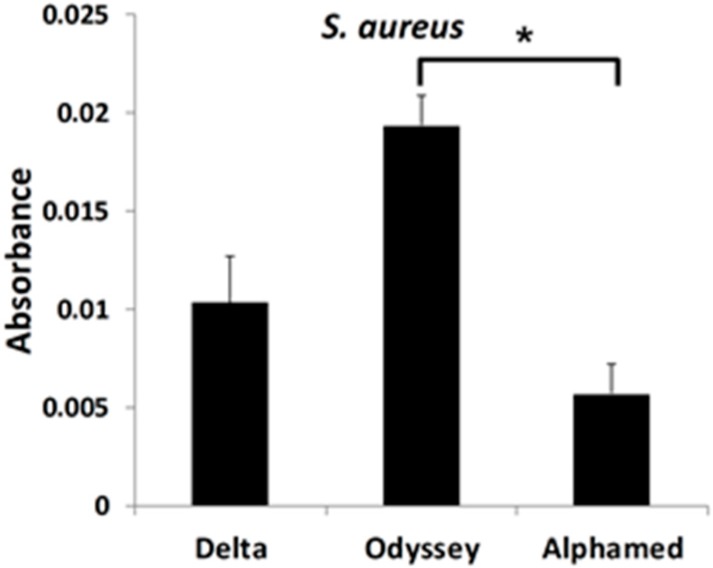
Quantitative measurements of bacterial growth on occluders. Occluders were placed individually in growing *S. aureus* for seven days. The presence of bacteria on occluders was determined by staining and elution with crystal violet. Absorbance of the eluted crystal violet was measured at an OD of 600 nm. * *p* < 0.05.

A ~3 fold statistically significant difference was detected in quantity of *S. aureus* between the Odyssey^R^ and Alphamed^R^ occluders. Although there was a higher trend (~2 fold) between the Odyssey^R^ and Delta^R^ occluders the data was not statistically significant (Figure 2).

### 3.2. S. aureus Preferentially Forms a Biofilm on Odyssey^R^ Occluder

**Figure 3:**
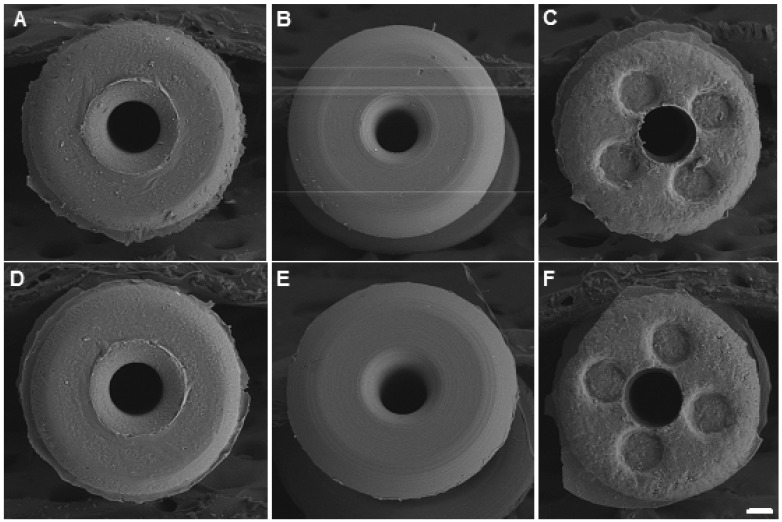
Image analyses of occluders. The top surface of the three types of unused (control) and with seven days in the presence of *S. aureus* (experimental) occluders were imaged using scanning electron microscopy (SEM). (**A**) Control Delta^R^; (**B**) Control Odyssey^R^; (**C**) Control Alphamed^R^; (**D**) Experimental Delta^R^; (**E**) Experimental Odyssey^R^ and (**F**) Experimental Alphamed^R^. Scale bar = 100 μm.

To visualize the presence of *S. aureus* and also to confirm the quantitative measurements, we employed SEM. Initially, we viewed the occluders under low magnification so that the entire surface could be examined. Figure 3 compares the top surface of each type of unused (scanned out of the box) occluder (Figure 3A–C) with the experimental ones (following a seven day incubation with bacteria) of the same type (Figure 3D–F). The Odyssey^R^ occluder appears to be the smoothest one (Figure 3B [control] and E [experimental]), followed by the Delta^R^ occluder, which also appears flat but not as smooth (Figure 3A [control] and D [experimental]). In contrast, the Alphamed^R^ occluder has a rougher surface and it contains four reservoir indentations (Figure 3C [control] and F [experimental]) designed to decrease foreign body sensation (5). No structural differences were observed at this magnification between the unused control (Figure 3A–C) and experimental (Figure 3D–F) occluders.

**Figure 4:**
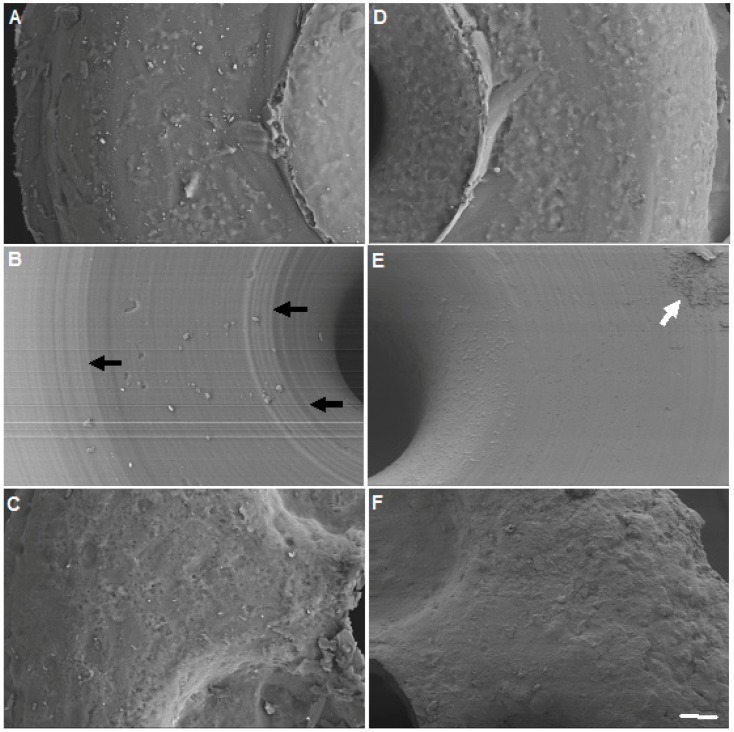
Surface and bacterial analyses of occluders. The top surface of the three types of unused (control) and with seven days in the presence of *S. aureus* (experimental) occluders were imaged using SEM. (**A**) Control Delta^R^; (**B**) Control Odyssey^R^; (**C**) Control Alphamed^R^; (**D**) Experimental Delta^R^; (**E**) Experimental Odyssey^R^ and (**F**) Experimental Alphamed^R^. Black arrows indicate grooves on the unused Odyssey^R^ occluder. White arrow indicates the presence of *S. aureus* biofilm on the Experimental Odyssey^R^ occluder. Scale bar = 20 μm.

This difference in appearance and smoothness between the three different types of occluders can be better appreciated in higher magnification images of the surface, as shown in Figure 4. Again, there is a clear difference between each of the three control types (Figure 4A–C) with the Odyssey^R^ being the smoothest of the three. Although it looks smoother, it is not completely flat, as circular grooves (reminiscent of vinyl records) are clearly visible (Figure 4B, black arrows), thus making this a rough surface. Additionally, these images also reveal the presence of bacterial colonization in the experimental occluders (Figure 4D–F), especially in the Odyssey^R^ and part of the *S. aureus* biofilm is indicated by the white arrow (Figure 4E).

**Figure 5:**
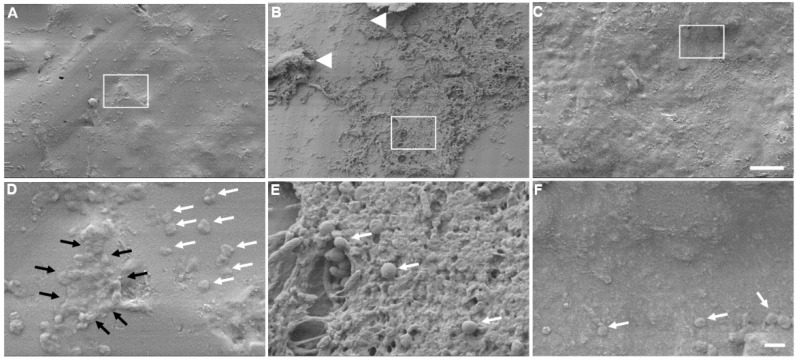
Biofilm formation. Surface morphology of the three types of experimental occluders (seven days in the presence of *S. aureus*) were imaged using SEM. (**A**,**D**) Delta^R^; (**B**,**E**) Odyssey^R^; (**C**,**F**) Alphamed^R^. Arrow heads indicate extensive three-dimensional (3D) biofilm formation observed with the Odyssey^R^ occluder. White arrows indicate the presence of individual *S. aureus* cells. Black arrows indicate the presence of a small biofilm present on the surface of the Delta^R^ occluder. White boxes in A, B, and C are areas magnified and shown in D, E, and F, respectively. Scale bar for A, B, C = 100 μm; Scale bar for D, E, F = 1 μm.

Higher magnification images, as shown in Figure 5, demonstrate the presence of individual bacteria in all three occluder types (Figure 5D–F, white arrows). The Odyssey^R^ occluder contained the greatest amount and extent of colonization by *S. aureus* (Figure 5B,E), thus confirming our quantitative measurements shown in Figure 2. An extensive and thick three-dimensional (3D) biofilm formed on this occluder (Figure 5B, white arrow heads and white box), as shown in even larger magnification in Figure 5E, where the 3D structural features of the biofilm can be clearly seen. Smaller areas of biofilm were also detected on the Delta^R^ occluder (Figure 5A). One such small area is shown in Figure 5D within the white box, revealing the early formation of a biofilm, as indicated by the surrounding black arrows, but again, its structural formation is less to that observed with the Odyssey^R^ occluder (Figure 5E). Moreover, the majority of bacteria were found in small clusters of two or three (Figure 5E, white arrows). Lastly, although bacteria were present on the Alphamed^R^ occluder (Figure 5F, white arrows), there was no detectable biofilm formation, just individual cells (Figure 5C,F, white arrows).

## 4. Discussion

The primary defense of most bacteria is the formation of a biofilm. *Staphylococcal* bacteria characteristically are found in biofilms. Because biofilms are found on any surface with moisture and nutrients present, ranging from freezing glaciers to boiling hot springs, it should not be surprising that they are found growing on occluders. In this study, we demonstrated the growth of *S. aureus* on all three occluder types. As bacterial infection is a rare but serious complication of punctal plug insertion, these findings lend credence to the possibility of occluders being the source for recurrent conjunctival and canalicular infections due to biofilm adhesion, and, in addition, may allow for a faster re-accumulation of biofilm on eyelids that have undergone a mechanical or electromechanical therapeutic lid scrub. In essence, the occluders would act as a nidus for biofilm reformation with cloistered colonies of *S. aureus*.

Previous studies demonstrated a strong causal relationship between *S. aureus* nasal colonization and increased risk of infection [11]. As the lacrimal duct connects to the nares, it is likely that this increased risk of infection extends to the tear duct. In a retrospective study evaluating the microbiologic spectrum of dacryocystitis, *S. aureus* was the most commonly cultured species, followed by *S. epidermitis* and *S. viridans*. Gram negative bacteria, such as *P. aeruginosa,* were far less common [13]. *S. aureus* and *S. epidermidis* are also the most frequent causes of nosocomial infection on indwelling medical devices [15]. Sugita et al. evaluated punctal occluders with SEM and cultured material extracted from plugs in 21 patients, finding positive culture results in 44%, isolating *S. epidermidis* in 75% of these and *S. aureus* in 25% [12]. Earlier, Yokoi et al. also found widespread bacterial colonization of *S. haemolyticus* and *C. tropicalis* on a removed punctal plug from a 63-year-old woman diagnosed as having tear-deficient type dry eye and treated with a punctal plug for several months [16]. Similarly, *P. aeruginosa* is notorious for causing chronic infection, particularly as the chief cause of corneal ulcer in contact lens wearers [17,18,19].

It is not clear why a statistically significant difference was found in the quantity of bacterial growth on the Odyssey^R^ occluder when compared with the Alphamed^R^. All of the occluders are made of silicone, and none were treated with any additional coatings. However, as the largest amount of bacteria was found on the Odyssey^R^, its smoother (al be it with grooves) texture does not appear to have affected biofilm formation. When comparing occluder shapes, the Odyssey^R^ has a collapsible nose that was designed to open inside the puncta, fitting the shape of the tear duct much like an umbrella unfolds (likely giving rise to its name, “Parasol”) [5]. It is possible that pooling of material within this umbrella-like cavity lead to a statistically significant difference in the quantity of bacteria when compared with the other two occluders. Further, all three punctal occluders were made of 100% silicone, but there was no documentation of their relative hydrophobicity or surface charge. Collectively, these factors are all known to play a role in bacteria-surface interactions.

Texture was considered as a differentiating factor and explored by SEM, revealing, at least qualitatively, the Odyssey^R^ as the smoothest but with circular grooves. The surprising evidence that the smoother surface occluder seemed to potentiate biofilm development might be explained by the mechanism of biofilm migration, which occurs via detachment, dispersal, rolling, and rippling. While one would not expect the detachment and dispersal to be affected by the roughness of the surface, rolling and rippling could indeed be affected and impeded by a rougher surface, similar to a hiker having more difficulty with a rough or mountainous terrain vs. a smooth flat terrain. Moreover, it is well established that bacterial adhesion is greater in grooved and braided materials as compared to flat ones due to increased surface area [20,21,22]. However, Katsikogianni and Missirlis [23] suggested that bacteria adhere preferentially to surface irregularities that resemble those of their size, since this maximizes bacteria-surface area. According to the authors, grooves or scratches that are on order of bacterial size increase the contact area and hence the binding potential [23]. It is conceivable then, that the Odyssey^R^ occluder with the visible regular grooves may serve as a desirable surface for *S. aureus* biofilm formation. Clearly, additional research is needed to further probe the exact size of the grooves that were observed on the Odyssey^R^ occluder.

There is new evidence that biofilm may play a significant role in dry eye disease with a unified theory that was proposed by Rynerson and Perry [2]. They suggest that bacterial biofilm coats the lid margin, creating inflammation that affects not only the Meibomian glands but also the lacrimal gland as well. This theory, known as DEBS (Dry Eye Blepharitis Syndrome), may lead to both evaporative and aqueous insufficiency, as the natural sequelae of decades of chronic blepharitis. Dry eye may be, in part, a late form and late manifestation of blepharitis. It is well known that bacteria colonize the lid margin with biofilm. The biofilm allows for population densities that initiate quorum-sensing gene activation. These newly activated gene products consist of inflammatory virulence factors, such as exotoxins, cytolytic toxins, and super-antigens. It may also be possible that punctal occluders may play a role in increasing inflammation to the lid margin by being a potent source of biofilm formation.

In conclusion, bacterial growth and biofilm is found on punctal occluders. The role of biofilm formation on these punctal occluders is not known but it may play a role in ocular surface inflammation. Unfortunately, there are not sufficient case reports or studies to prove a clinical difference between these three occluders. In addition, there is no evidence, case reports or studies to prove a differential exists clinically between these three occluders. As this was only an *in vitro* study, further evaluation of punctal occluders in relation to bacterial adherence, biofilm formation, as well as DED in vivo is warranted.

## References

[1] Lemp M.A., Baudouin C., Baum J., Dogru M., Foulks G.N., Kinoshita S., Laibson P., McCulley J., Murube J., Pflugfelder S.C. (2007). The definition and classification of dry eye disease: Report of the Definition and Classification Subcommittee of the International Dry Eye WorkShop. Ocul. Surf..

[2] Rynerson J.M., Perry H.D. (2016). DEBS—A unification theory for dry eye and blepharitis. Clin. Ophthalmol..

[3] O’Brien P.D., Collum L.M.T. (2004). Dry eye: Diagnosis and current treatment strategies. Curr. Allergy Asthma Rep..

[4] Tost F., Geerling G., Geerling G., Brewitt H. (2008). Plugs for occlusion of the lacrimal drainage system. Surgery for the Dry Eye.

[5] Jehangir N., Bever G., JafarMahmood S.M., Moshirfar M. (2016). Comprehensive review of the literature on existing punctal plugs for the management of dry eye disease. J. Ophthalmol..

[6] Horwath-Winter J., Thaci A., Gruber A., Boldin I. (2007). Long-term retention rates and complications of silicone punctal plugs in dry eye. Am. J. Ophthalmol..

[7] Costerton J.W., Geesey G.G., Cheng K.J. (1978). How bacteria stick. Sci. Am..

[8] Limoli D.H., Jones C.J., Wozniak D.J. (2015). Bacterial extracellular polysaccharides in biofilm formation and function. Microbiol. Spectr..

[9] Edwards A.M., Bowden M.G., Brown E.L., Laabei M., Massey R.C. (2012). *Staphylococcus aureus* extracellular adherence protein triggers TNFα release, promoting attachment to endothelial cells via protein A. PLoS ONE.

[10] Sanchez-Vizuete P., Orgaz B., Aymerich S., Le Coq D., Briandet R. (2015). Pathogens protection against the action of disinfectants in multispecies biofilms. Front. Microbiol..

[11] Archer N.K., Mazaitis M.J., Costerton J.W., Leid J.G., Powers M.E., Shirtliff M.E. (2011). *Staphylococcus aureus* biofilms: Properties, regulation, and roles in human disease. Virulence.

[12] Sugita J., Yokoi N., Fullwood N.J., Quantock A.J., Takada Y., Nakamura Y., Kinoshita S. (2001). The detection of bacteria and bacterial biofilms in punctal plug holes. Cornea.

[13] Eshraghi B., Abdi P., Akbari M., Fard M.A. (2014). Microbiologic spectrum of acute and chronic dacryocystitis. Int. J. Ophthalmol..

[14] Laibson P.R., Donnenfeld E.D. (1986). Corneal ulcers related to contact lens use. Int. Ophthalmol. Clin..

[15] Otto M. (2008). Staphylococcal biofilms. Curr. Top. Microbiol. Immunol..

[16] Yokoi N., Okada K., Sugita J., Kinoshita S. (2000). Acute conjunctivitis associated with biofilm formation on a punctal plug. Jpn. J. Ophthalmol..

[17] Mayo M.S., Cook W.L., Schlitzer R.L., Ward M.A., Wilson L.A., Ahearn D.G. (1986). Antibiograms, serotypes, and plasmid profiles of Pseudomonas aeruginosa associated with corneal ulcers and contact lens wear. J. Clin. Microbiol..

[18] Miller M.J., Ahearn D.G. (1987). Adherence of Pseudomonas aeruginosa to hydrophilic contact lenses and other substrata. J. Clin. Microbiol..

[19] Glastonbury J., Crompton J.L. (1989). Pseudomonas aeruginosa corneal infection associated with disposable contact lens use. Aust. N. Z. J. Ophthalmol..

[20] Scheuerman T.R., Camper A.K., Hamilton M.A. (1998). Effects of substratum topography on bacterial adhesion. J. Colloid Interface Sci..

[21] Bos R., van der Mei H.C., Gold J., Busscher H.J. (2000). Retention of bacteria on a substratum surface with micropatterned hydrophobicity. FEMS Microbiol. Lett..

[22] Medilanski E., Kaufmann K., Wick L., Wanner O., Harms H. (2002). Influence of surface topography of stainless steel on bacterial adhesion. Biofoul.

[23] Katsikogianni M., Missirlis Y.F. (2004). Concise review of mechanisms of bacterial adhesion to biomaterials and of techniques used in estimating bacteria-material interactions. Eur. Cell Mater..

